# Pilot study of an internet-based pain coping skills training program for patients with systemic Lupus Erythematosus

**DOI:** 10.1186/s41927-021-00191-6

**Published:** 2021-06-17

**Authors:** Kelli D. Allen, Tyler Beauchamp, Christine Rini, Francis J. Keefe, Kim L. Bennell, Rebecca J. Cleveland, Kimberlea Grimm, Katie Huffman, David G. Hu, Andres Santana, Shruti Saxena Beem, Julie Walker, Saira Z. Sheikh

**Affiliations:** 1 Department of Medicine, Division of Rheumatology, Allergy and Immunology, University of North Carolina at Chapel Hill, Chapel Hill, NC USA; 2 Thurston Arthritis Research Center, School of Medicine, University of North Carolina at Chapel Hill, Chapel Hill, NC USA; 3 Center of Innovation to Accelerate Discovery and Practice Transformation, Department of Veterans Affairs Health Care Center, Durham, NC USA; 4 Medical College of Georgia, Augusta, GA USA; 5 Department of Medical Social Sciences, Feinberg School of Medicine, Northwestern University, Chicago, IL USA; 6 Robert H. Lurie Comprehensive Cancer Center, Northwestern University, Chicago, IL USA; 7 Department of Psychiatry and Behavioral Sciences, Duke University School of Medicine, Durham, NC USA; 8 Centre for Health, Exercise, and Sports Medicine, Department of Physiotherapy, University of Melbourne, Melbourne, VIC Australia

**Keywords:** Systemic lupus Erythematosus, Coping, Pain

## Abstract

**Background:**

Patients with Systemic Lupus Erythematosus (SLE) often experience pain and other symptoms that negatively impact quality of life. Interventions that enhance the use of behavioral and cognitive coping strategies may lead to improved outcomes among patients with SLE. Pain coping skills training (PCST) programs have been shown to improve outcomes among patients with other rheumatic conditions, but there have been no trials of PCST among patients with SLE. This study was a preliminary assessment of the feasibility and efficacy of painTRAINER, an automated, internet-based PCST program, among patients with SLE.

**Methods:**

Participants (*n* = 60) with SLE from one health care system were randomly assigned with equal allocation to painTRAINER or a wait list control group. PainTRAINER involves 8 modules; participants were instructed to complete one module weekly, along with practice activities for each cognitive or behavioral coping skill. Outcome measures were assessed at baseline and 9-week follow-up, including the Pain Catastrophizing Scale, PROMIS Subscales (Pain Interference, Physical Function, Sleep Disturbance, Anxiety, Depression, Fatigue and Participation), and the LupusPRO questionnaire. Mean changes in outcomes from baseline to follow up and Cohen’s d effect sizes were computed.

**Results:**

Effect sizes for the painTRAINER group (relative to the wait list group) were small, with changes being greatest for the PROMIS Depression score (*d* = − 0.32). Among those randomized to the painTRAINER group, 50% accessed the program (“painTRAINER users”). Most of those who did not access the program stated that they did not receive instructions via email. Effect sizes for “painTRAINER users” (relative to wait list) were larger than for the whole painTRAINER group: Pain Catastrophizing *d* = − 0.60, PROMIS Pain Interference *d* = − 0.3., PROMIS Depression *d* = − 0.44, LupusPRO Health-Related Quality of Life *d* = 0.30.

**Conclusions:**

PainTRAINER users reported meaningful improvements in multiple physical and psychological outcomes, supporting the potential of PCST programs to benefit individuals with SLE. However, strategies are needed to improve engagement with the program and tailor content to comprehensively address key SLE symptoms and challenges.

**Trial registration:**

NCT03933839, May 1, 2019.

## Background

Systemic lupus erythematosus (SLE) is a chronic autoimmune disease that impacts multiple organ systems. Individuals with SLE often experience pain and a variety of health-related challenges including fatigue, anxiety and depressive symptoms, and disability [1]. SLE is associated with flare-ups and periods of remission that are unpredictable, making it a complex disease to manage clinically [2]. Due to the relatively young average age of SLE onset, many patients must navigate these challenges while maintaining work and/or caring for young children. Because of these factors, SLE often has a major impact on patients’ quality of life.

Prior studies show that greater use of pain coping efforts and greater self-efficacy for coping with SLE-related symptoms are associated with better physical and psychological outcomes (i.e. fatigue, pain, psychological distress, health-related quality of life) [3–7]. Also, pain catastrophizing (i.e. focusing on and exaggerating the threat of pain and negatively evaluating one’s ability to deal with pain) has been associated with poorer SLE outcomes [3–7]. Importantly, many studies in other rheumatic conditions have shown that pain coping skills training (PCST) programs can improve coping patterns as well as physical and psychological health outcomes [8–11]. However, there have been no trials of PCST among individuals with SLE, who face a unique set of disease-related challenges (i.e. recurring and remitting symptoms, unpredictable disease course, wide range of symptoms and complications) and are younger on average than many with other rheumatic conditions. Delivery of PCST programs to patients with SLE could have a positive impact on outcomes and quality of life, but this evidence base needs to be established. A few small trials have examined psychological interventions that incorporate aspects of pain coping, showing positive impacts on both psychological and physical health measures in patients with SLE [12–16]. Although these studies show promise for coping skills interventions as a component of SLE care, most previously studied interventions involve multiple in-person visits or group sessions, which can limit reach and participation. There is a need for coping skills interventions that are widely and easily accessible to patients with SLE. To address this important gap, we conducted a pilot study of an automated, freely available, internet based PCST program, painTRAINER, which has been shown to improve multiple key outcomes among patients with osteoarthritis [17]. Specifically, we evaluated the feasibility and acceptability of painTRAINER among patients with SLE and conducted a preliminary assessment of efficacy. We chose to study painTRAINER because pain is a commonly reported symptom among individuals with SLE and because the cognitive and behavioral coping skills included in this intervention can apply to other symptoms and health-related challenges.

## Methods

### Study design

We conducted a randomized pilot and feasibility study, with equal allocation of patients to painTRAINER and a wait list control group in which participants were offered painTRAINER after completion of follow-up assessments. Outcomes were assessed at baseline and 9-week follow-up, similar to Rini et al. [17], as painTRAINER is designed for delivery over 8 weeks. Baseline and follow-up questionnaire data were collected via telephone by a trained study team member; follow-up assessments were conducted by blinded study team members only. Participants were paid $25 for completion of assessments at each time point. Participants were permitted to continue with all usual SLE care during study participation. The study was approved the University of North Carolina at Chapel Hill (UNC) Institutional Review Board. Methods were carried out in accordance with relevant guidelines and regulations.

### Participants and enrollment

Participants were 60 patients age ≥ 18 with SLE from UNC Health Care System. We used the UNC Carolina Data Warehouse for Health (CDW-H) to identify potential participants using date of birth and ICD-10 codes for SLE (M32.x), then conducted a brief chart review to verify diagnosis of SLE. The Carolina Data Warehouse for Health (CDW-H) is a central data repository containing clinical, research, and administrative data sourced from the UNC Health Care System. Both current and legacy hospital systems are represented, with the ability to query most data elements as far back as mid-2004; for this study, we identified patients with codes for SLE from April 2018–April 2019.

Potentially eligible patients were mailed a recruitment letter (including an opt-out telephone number), followed by a telephone call about 1 week later to assess eligibility and interest. Rheumatology providers could also refer patients with SLE to the study team for screening. During the screening call, we assessed the patient’s internet access and availability of a device on which they could use painTRAINER (computer, tablet, smartphone); for this study, patients were excluded if they could not access painTRAINER to complete the intervention. Next, participants were asked to rate their current, generalized pain on a scale of 0–10; we required a score of at least 4 for study inclusion, similar to other studies [18, 19]. Exclusion criteria, assessed from the medical record and phone screening, included: significant memory loss, active psychosis or substance abuse, neuropsychiatric SLE, severe hearing impairment, inability to speak English, pregnant or planning to become pregnant in the next 3 months, and current participation in another SLE-related trial. After screening eligibility was determined, participants provided verbal consent and HIPAA authorization. Participants then completed baseline assessments and were given their randomization assignment, based on a computer-generated sequence developed by the study statistician. All study data was stored using Research Electronic Data Capture, or REDCap, a secure web application that can be used to build and manage case report forms, surveys and other data capture mechanisms for clinical research [20].

### PainTRAINER intervention

PainTRAINER is an eight-week, automated (i.e., self-completed, without therapist involvement), internet-based version of PCST [17]. The training it provides is based on a therapist-delivered PCST program that has a strong evidence base for improving coping efforts, pain interference, and other pain-related outcomes in patients with chronic pain-related conditions [8–11]. PainTRAINER was rigorously designed to retain key therapeutic features of therapist-delivered PCST, while presenting training in an easy-to-use format that includes guided feedback, interactive exercises and animated demonstrations [18].

Following randomization, participants assigned to painTRAINER were given a verbal overview of the program and were sent, via email (or mailed a hard copy if requested), instructions for accessing the program, unique login information, and study team contact information in case of questions or technical difficulties. Participants were called about 1 week after these initial instructions as a reminder to log into painTRAINER and check for any technical difficulties. After participants begin the program, painTRAINER sends automated email reminders to practice skills and complete modules. (One person in this study chose not to provide a personal email, so the study team sent these reminders via mail.) Participants were instructed to begin using painTRAINER immediately and encouraged to complete all modules before the 9-week follow-up assessment. PainTRAINER includes eight modules to be completed at a rate of one per week [17]; content is shown in Table 1. Each module takes about 30–45 min to complete. During each module, a “virtual coach” greets participants and provides verbal instructions about a specific coping skill and practicing skills. Key points for each module are also highlighted with on-screen text and / or graphics, and the program helps participants develop coping skills with interactive exercises that include guided practice and evaluating their experience with them. Modules and features of painTRAINER are customized to participants using computer- algorithms based on their responses and progress through the program.

**Table 1:** PainTRAINER Modules

#	Coping Skill(s)	Content
1	Progressive Muscle Relaxation	• Introduce program and concept of pain coping skills • Therapeutic rationale (how thoughts, feelings, and actions affect pain through pain “gate”) • Introduce progressive muscle relaxation • Exercise: Provide opportunity to practice progressive muscle relaxation • Help user identify positive aspects of experience to reinforce use of skill • Help user identify and address barriers to use of skill • Describe importance of regular practice and how to set up practice reminders • Set practice goal
2	Mini Relaxation Practices	• Review progressive muscle relaxation and practices completed in prior week • Introduce “mini-practices” (brief relaxation) • Exercise: Provide an opportunity to do sitting and standing mini-practices • Help user identify positive aspects of experience to reinforce use of skill • Help user identify and address barriers to use of skill • Describe how to set up practice reminders and set practice goal
3	Activity / Rest Cycling	• Review mini-practice and practices completed in prior week • Introduce activity/rest cycling • Exercise: Identify activities user tends to overdo • Vicarious learning: Demonstrate how others have changed overdone activities • Exercise: Create personal plan to use skill that fits personal activities and goals • Discuss how other skills help with use of this one • Set practice goals for this skill and review practice goals for other skills
4	Pleasant Activity Scheduling, Negative Automatic Thoughts	• Review activity/rest cycling and practices completed in prior week • Introduce pleasant activity scheduling • Exercise demonstrating how to select and add pleasant activities to routine • Schedule 3 pleasant activities for week • Problem-solve barriers with interactive vicarious learning exercise • Introduce concept of negative automatic thoughts • Exercise demonstrating how to identify negative automatic thoughts • Set practice goals for week and review practice goals for other skills
5	Negative Automatic Thoughts, Coping Thoughts	• Review pleasant activity scheduling and practices completed in prior week • Continue lesson on automatic thoughts, then introduce concept of coping thoughts • Exercise: Identifying negative thoughts and reactions to them • Exercise: Creating coping thoughts to address negative thoughts • Exercise: Identify and address circumstances that hinder use of skill • Set practice goals for week and review practice goals for other skills
6	Pleasant Imagery and other Distraction Techniques	• Review coping thoughts and practices completed in prior week • Introduce pleasant imagery and other distraction techniques • Provide an opportunity to practice pleasant imagery and explore experience • Exercise on identifying negative thoughts and reactions to them • Set practice goals for week and review practice goals for other skills
7	Problem Solving	• Review pleasant imagery, distraction, and practices completed in prior week • Introduce concept of problem solving • Demonstrate problem solving, illustrated by stories told by other people • Exercise: Select skills for different situations, with personal plan for overcoming barriers • Set practice goals for week and review practice goals for other skills
8	Monitoring for Maintenance	• Review content of all modules • Exercise: Evaluate skill use and helpfulness, including comparison with others’ experiences • Exercise: Develop plan for maintaining use of skills • Present rationale to motivate continued practice and skill development • Review practice goals for skills

### Measures

#### Feasibility and acceptability measures

Feasibility metrics included proportions of eligible and enrolled patients, proportion of completed follow-up assessments, reasons for ineligibility and dropout, and number of participants accessing the painTRAINER program. We also asked for participants’ feedback about the intervention following completion. This feedback included an overall rating of the helpfulness of painTRAINER with respect to managing or coping with SLE symptoms (0 = Not at all helpful to 10 = Very helpful), ratings of the helpfulness of each coping skill addressed (0 = Not at all helpful to 10 = Very helpful), and an open-ended question asking participants for recommendations for improving painTRAINER. We obtained data from the painTRAINER program regarding modules completed by each participant.

#### Efficacy measures

##### PROMIS pain interference instrument (form 6a)

This 6-item, validated measure assesses self-reported consequences of pain across aspects of life including social, cognitive, emotional, physical and recreational activities [19]. Items are scored on a Likert scale of 1 (not at all) to 5 (very much), with higher scores indicating greater pain interference.

##### PROMIS-29

This 29-item scale covers 7 domains of self-reported health: Depression, Anxiety, Physical Function, Pain Interference, Fatigue, Sleep Disturbance, and Ability to Participate in Social Roles and Activities [21]. All items are scored using a 5-point Likert format, with higher scores indicating more of that domain; therefore, higher scores indicate a more positive outcome for Physical Function and Ability to Participate and a more negative outcome for the other domains.

##### Coping strategies questionnaire (secondary outcome)

This 48-item scale covers 7 domains: Catastrophizing, Diverting Attention, Ignoring Sensations, Coping Self-Statements, Reinterpreting Pain Sensations, Praying-Hoping, Increasing Behavioral Activities [22, 23]. Consistent with other studies [24, 25], we calculated a Coping Attempts Score, which summed all domains other than Catastrophizing.

##### LupusPRO (secondary outcome)

LupusPRO (v1.8) is a 43-item reliable, validated self-report measure including domains of Lupus Symptoms, Lupus Medication, Physical Health, Emotional Health, Pain, Sleep, Procreation, Cognition, Body Image, Desires-Goals, Coping, Social Support and Satisfaction with Care [26]. We assessed separate domains, as well as health-related quality of life and non-health-related quality of life subscales that combined domains.

#### Demographic and clinical characteristics

We assessed self-reported age, sex, race / ethnicity, household financial status (living comfortably or just meeting basic expenses with a little left over for extras vs. just meeting basic expenses or don’t have enough to meet basic expenses), education level (no college vs at least some college education), marital status (married or living with a partner as married vs. other), work status (working part or full time vs. other), children (and their ages), body mass index, duration of SLE symptoms and time since diagnosis, self-rated health, and comorbid illnesses [27].

### Statistical analysis

We calculated descriptive statistics for covariates and measures at baseline, presenting continuous variables as means and standard deviation (SD) and categorical variables as percentages. Feasibility and acceptability metrics were assessed with simple descriptive statistics such as proportions and means or medians with corresponding distributions. Responses to open-ended questions regarding painTRAINER experiences and recommendations were compiled and grouped by theme. We calculated means of all outcomes at both time points, as well as mean changes and associated 95% confidence intervals between baseline and follow-up for painTRAINER and control groups. Because of the small sample size, we did not conduct formal statistical testing. However, we calculated Cohen’s *d* effect sizes as a preliminary metric of efficacy. Analyses were conducted using SAS statistical software version 9.4 (SAS Institute Inc., Cary, NC).

## Results

### Participants, feasibility and acceptability metrics

**Fig. 1:**
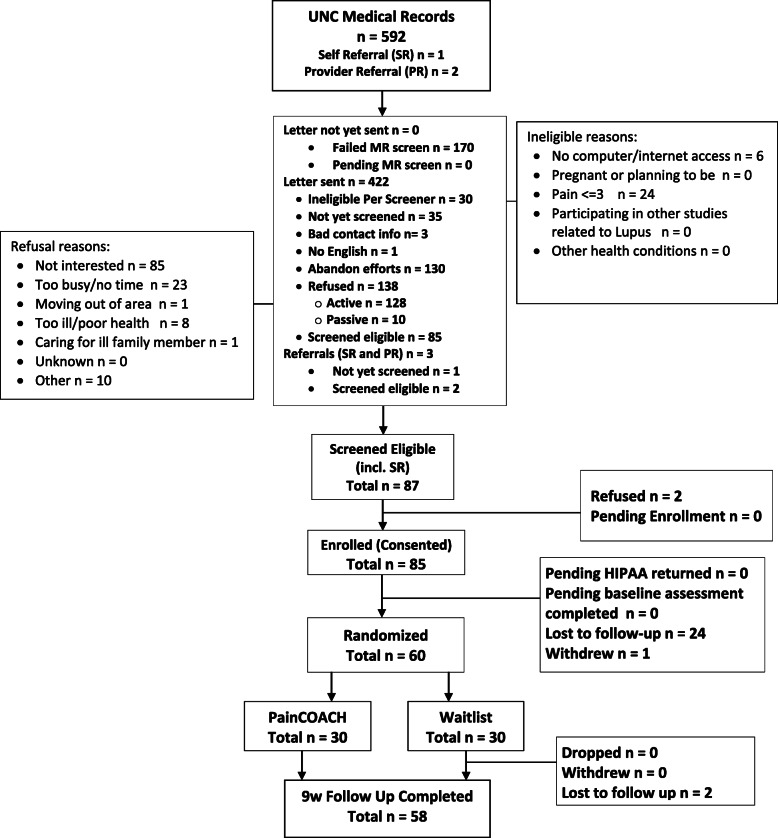
PainTRAINER consort flowchart

We identified 592 potentially eligible patients from the UNC electronic health record; 2 additional individuals were referred by a health care provider and 1 self-referred (Fig. 1). Of these 595, 87 screened eligible and 85 verbally consented to participate via telephone. Reasons for ineligibility and refusal prior to screening are shown in Fig. 1. Of the 85 who consented, 24 were lost to follow-up and 1 withdrew prior to randomization, leading to the final sample size of 60 participants. Among the 60 participants randomized, 58 completed follow-up assessments (29 in the painTRAINER group, 29 in the Wait List control group). Characteristics of study participants are shown in Table 2.

**Table 2:** Participant Characteristics

	Wait List (*N* = 30)	painTRAINER (*N* = 30)	painTRAINER Users (*N* = 15)
**Mean Age (SD)**	47 (12)	51 (14)	51 (14)
**Mean # of Children (SD)**	1.4 (1.1)	1.6 (1.4)	1.5 (1.6)
**Mean Age of Children (SD)**	22 (10)	32 (10)	29 (10)
**Mean Yrs w/ Lupus (SD)**	14 (11)	16 (16)	17 (21)
**% With Self-Reported Excellent, Very Good or Good Health**	37%	37%	40%
**Mean # of Comorbidities**	3.4 (2.5)	3.3 (2.2)	3.0 (2.4)
**% Female**	93%	97%	100%
**% Caucasian**	37%	33%	33%
**% With Some College Education**	87%	77%	80%
**% Married / Living with Partner**	50%	40%	40%
**% With “Adequate” Income**	83%	72%	80%
**% Working**	37%	23%	23%

Among participants randomized to the painTRAINER group, *n* = 15 (50%) accessed the program (“painTRAINER users”). Most of those who did not access the program (*n* = 9) stated they did not receive instructions via email or did not know they were supposed to proceed with accessing the program; 2 additional participants stated that they had technical difficulties and could not log on to the program. The median rating of helpfulness of painTRAINER (on a scale of 0–10), from those who used the program, was 8.0. Median ratings of the specific modules ranged from 6.5 (Understanding Pain and Relaxation, Pleasant Activity Scheduling) to 8.0 (Activity / Rest Cycle, Pleasant Imagery, Problem Solving, Looking Back and Moving Forward).

Participants’ responses regarding feedback on painTRAINER focused on two major areas. First, participants consistently praised the convenience of the online format, that you can “do it on your own schedule” when you “have time to focus” and that “you didn’t have to drive” to do the program. Second, some participants noted that it was difficult for them to relate to some aspects of painTRAINER. For example, one participant stated that “some of the descriptions of pain were not accurate” for people with lupus. Another participant noted that their pain “has nothing to do with my activities.”

### Efficacy outcomes

Table 3 shows changes in all study outcomes (follow-up minus baseline) for painTRAINER and wait list groups. Because a substantial number of participants did not log into the program, we also present data specifically for the painTRAINER users. PainTRAINER users had a small decrease in mean Pain Catastrophizing score, while participants in the other groups (e.g., full painTRAINER group and wait list group) had increases in mean Pain Catastrophizing scores; the effect size for painTRAINER users, relative to the Wait List group was − 0.60. Participants in the full painTRAINER group had greater improvement in PROMIS Pain Interference than the wait list group, with painTRAINER users having the greatest improvement (effect size − 0.30 compared with the Wait List group). All groups had decreases in PROMIS Physical Function scores. The full painTRAINER group had greater improvements in PROMIS Depression scores than the Wait List Group (effect size = − 0.34). PainTRAINER users also had more favorable responses than the Wait List group with respect to PROMIS sleep disturbance, anxiety, fatigue and the LuposPRO Health-Related Quality of Life Score.

**Table 3:** Mean Changes (SD), Follow-up minus Baseline, and Effect Sizes Compared to Wait List

	Wait List (***N*** = 30) Mean (SD) Change	painTRAINER (***N*** = 30)		painTRAINER Users (***N*** = 15)	
		Mean (SD) Change	Effect Size	Mean (SD) Change	Effect Size
**Pain Catastrophizing**^**b**^	3.6 (6.5)	2.3 (9.6)	−0.16	−0.9 (8.9)	−0.60
**PROMIS Pain Interference**^**b**^	−1.7 (790)	−2.6 (6.6)	− 0.12	−3.9 (6.5)	− 0.30
**PROMIS Physical Function**^a^	− 0.6 (7.0)	− 3.8 (4.1)	− 0.56	−4.0 (4.4)	− 0.55
**PROMIS Participation**^a^	0.5 (6.8)	0.7 (6.5)	0.03	0.8 (7.4)	0.05
**PROMIS Sleep Disturbance**^**b**^	0.4 (9.3)	0.6 (9.4)	0.02	−2.1 (7.8)	−0.28
**PROMIS Anxiety**^**b**^	0.4 (11.2)	1.4 (9.3)	0.09	−0.7 (8.1)	−0.11
**PROMIS Depression**^**b**^	−0.6 (9.0)	−3.4 (8.6)	−0.32	−4.1 (5.2)	− 0.44
**PROMIS Fatigue**^**b**^	−3.5 (7.1)	−2.2 (8.2)	0.17	−5.1 (6.2)	−0.23
**LupusPRO Health-Related Quality of Life Score**^c^	1.4 (12.0)	2.0 (13.3)	0.04	4.9 (11.1)	0.30
**LupusPRO Non-Health-Related Quality of Life Score**^**d**^	3.7 (13.4)	3.9 (13.3)	0.02	3.1 (14.7)	−0.04

^a^Positive score indicates improvement

^b^Negative score indicates improvement

^c^Includes physical health, pain, symptoms, cognition, medications and procreation

^d^Includes satisfaction with care, coping, desires-goals, and social support

## Discussion

In this pilot study, we found preliminary support that painTRAINER may improve key outcomes, including pain catastrophizing, pain interference, and depressive symptoms, among individuals with SLE who use the program. These are encouraging results given the low cost of disseminating this type of automated, internet-based intervention and the potential for widespread use. However, the study also revealed some challenges with study recruitment, as well as engaging patients in use of the painTRAINER program, which will need to be addressed in future research.

Regarding study recruitment, among patients to whom we mailed recruitment letters (*n* = 422), about 64% either did not respond to our recruitment calls or declined participation (Fig. 1). This proportion is similar to other studies [24, 28, 29]. However, we believe there are additional recruitment strategies that could improve recruitment in a future study. In this preliminary study we proactively reached out to patients with SLE in one health care system. This type of recruitment strategy can lead to a more generalizable study sample compared with studies that rely only on self-referral. However, self-referral recruitment strategies offer the advantage of reaching individuals who are particularly interested in the intervention and therefore more likely to participate in the study and engage with the program. In a future study, we plan to continue a proactive recruitment strategy but also utilize multiple strategies (e.g. advertisements, social media) to encourage self-referrals to the study. Motivational interviewing and other orientation strategies may also help to foster engagement among those recruited [30, 31].

Among patients we contacted who did not enter the study, the primary reason was that people were not interested or too busy; this reinforces the notion that adding efforts to increase self-referrals may be helpful in a future study. Some participants (*n* = 24) were ineligible due to low pain levels. We included this criterion because of painTRAINER’s focus on pain. However, we recognize there are other symptoms and health challenges commonly faced by patients with SLE that painTRAINER could address, outside of pain. In a future study, we plan to modify the program so it addresses a broader set of challenges; inclusion criteria would be modified accordingly. Modifying the program will also allow us to address participant feedback that the current version of the program was not always optimally relevant to the type of pain and other symptoms they experienced. There were also some individuals (*n* = 6) who did not have internet access and therefore could not participate. Potential solutions to address this issue include modifying the program so that it is easily accessible via phone and providing participants with internet access and/or mobile devices to use painTRAINER. We also note that study participants had, on average, a relatively long duration of SLE (about 15 years), and the average age was about 45 years. This may impact generalizability of findings. In addition, individuals with longer-standing disease may have more established cognitive behavioral patterns that are more difficult to change; additional efforts are needed to understand how we can more effectively reach individuals with this type of the program earlier in the disease course.

The proportion of participants who completed follow-up assessments was high (97%) and similar to other studies in this area, which have ranged from 90 to 96% [24, 28]. However, a key issue in this study was that only 16 of the 30 individuals in the painTRAINER group accessed the program. This level of engagement is lower than in prior studies [17, 24, 28]. Among participants who did not access painTRAINER, 7 said they did not receive an email with instructions for accessing the program, 4 stated they did not know they were supposed to access the program, and 2 had technical difficulties. Although we had a process for calling participants within about a week of sending the email to ensure they received the information and could access the painTRAINER program, we were unable to reach some patients via phone. It is possible that some participants indicated they had not received the information (or did not know they were supposed to access the program) due to “social desirability” – e.g., not wanting to tell a study team member that they chose not to access the program or were not interested. This possibility further stresses the importance of employing strategies to reach out to patients who are most interested in and perceive a need for this type of program to help them with managing their SLE symptoms. However, in a future study we will utilize a range of methods, based on participants’ communication preferences (e.g., text, mail, hone, email) to ensure patients receive instructions for accessing painTRAINER. We believe a process in which individuals can provide consent, complete baseline measures, and begin accessing the program all within the same platform will facilitate program engagement, as well as recruitment. We also acknowledge that the low level of engagement may signal that changes are needed to make painTRAINER content of greater interest to patients with SLE.

Ratings of the helpfulness of painTRAINER were relatively high (8.0 on a scale of 0–10), which suggests that users found the program beneficial. This rating was the same as reported in our recent study of a telephone-based, counselor-delivered pain coping skills training intervention for African Americans with osteoarthritis [32]. We believe the helpfulness of the program can be increased by expanding it to include other symptoms and challenges common among individuals with SLE. Participants’ responses to open-ended feedback questions suggested that this was a primary concern.

Overall, this preliminary study provides support for the efficacy of painTRAINER among patients with SLE. Pain catastrophizing, as well as PROMIS pain interference, sleep disturbance, fatigue anxiety, and depression all improved more in painTRAINER users compared to the wait list group, with the largest improvements being in Pain Catastrophizing (− 0.60 effect size). This is important given the negative impacts of pain catastrophizing on a variety of health-related outcomes among patients with SLE and other rheumatic conditions [4, 33–35]. It is interesting that the painTRAINER group had greater improvements in the LupusPRO health-related mean quality of life score compared with the wait list group, while there was not a substantial between-group differences in the non-health related mean quality of life score. These two composite scores are a mix of domains, and it is possible that painTRAINER differentially impacted some of the individual domains within the composite scores. However, the individual domains have limited numbers of items and scale ranges, and with the small sample size in this study, it was not feasible to compare groups on the individual domains.

There were several limitations to the study. Because this was a small pilot study, generalizability may be limited. Along with this, participants were selected from one academic medical center. A future trial would include a larger sample recruited from a broader range of settings and data sources. The duration of follow-up assessment was limited to 9 weeks, immediately following completion of the intervention period. Although this is reasonable for a preliminary study, a larger study will need to examine longer term outcomes. Finally, since painTRAINER is available as a free program to the public, it is possible that individuals in the control group utilized the program during their study period. However, we believe this is relatively unlikely since this program has not been widely publicized in the U.S., and we did not provide information on the program to Wait List participants until after their follow-up assessments were completed.

## Conclusions

In summary, this study provided a valuable preliminary assessment of painTRAINER among patients with SLE. We have identified several important considerations for a future, larger effectiveness trial. First, since painTRAINER was not developed specifically for patients with SLE, we need to tailor the program for this patient group, addressing key issues including SLE-specific pain symptoms, fatigue, treatment related challenges, comorbidities, and work and caregiving related limitations. Our next step to address this is to work with patient stakeholders to develop strategies for incorporating SLE-related content into painTRAINER. Second, as noted above, we need to develop broader recruitment strategies, potentially including multiple clinical sites, SLE registries, advocacy organizations, and social media. Third, a potential strategy to foster engagement with the intervention is to include a “real time” virtual component involving peer groups and / or a trained facilitator. Although the convenience of the online painTRAINER format was very appealing to participants in this study, adding the support and accountability of participating in the program alongside others may boost engagement and efficacy. Therefore, our study team and patient stakeholders will consider potential real-time delivery strategies within the study design.

## Data Availability

The full data sets generated during the current study are not publicly available because they include personally identifiable data from study participants. However, deidentified data are available from the corresponding author on reasonable request.
